# Comparative Transcriptomic and Physiological Profiling Reveals Species-Associated Differences Across Five Duckweed Species

**DOI:** 10.3390/ijms27219681

**Published:** 2026-10-29

**Authors:** Doni Thingujam, Zhengzhi Tan, Yiqing Wang, Karolina M. Pajerowska-Mukhtar, M. Shahid Mukhtar

**Affiliations:** 1 Department of Biological Sciences, Clemson University, 132 Long Hall, Clemson, SC 29634, USA; dthingu@clemson.edu (D.T.); kmukhta@clemson.edu (K.M.P.-M.); 2 Department of Genetics & Biochemistry, Clemson University, 105 Collings St., Biosystems Research Complex, Clemson, SC 29634, USA; tzhengz@clemson.edu (Z.T.); yiqingw@clemson.edu (Y.W.)

**Keywords:** Lemnaceae, monocot, RNA sequencing, de novo assembly, orthogroup, gene ontology, nitrogen, chlorophyll, peroxidase

## Abstract

Duckweeds (Lemnaceae) are fast-growing aquatic monocots with potential for biomass production and phytoremediation, but baseline molecular and physiological differences among species remain poorly characterized. We assembled de novo transcriptomes of five duckweed species, *Lemna minor*, *Lemna japonica*, *Lemna gibba*, *Landoltia punctata*, and *Lemna aequinoctialis*, grown on basal Murashige and Skoog (MS) medium (three biological replicates per species), and evaluated for completeness with Benchmarking Universal Single-Copy Orthologs (BUSCO) against Viridiplantae, Embryophyta, and Liliopsida reference sets. Orthogroup-level expression divergence was characterized by species signature scoring and Pearson correlation analysis; Gene Ontology (GO) and Kyoto Encyclopedia of Genes and Genomes (KEGG) annotations were examined using descriptive composition analyses and formal overrepresentation tests. In an independent experiment, biomass, protein content, glucose accumulation, peroxidase activity, and chlorophyll a/b concentrations were measured after 7 days under four nutrient regimes (Murashige and Skoog (MS) medium, Hoagland solution, Gamborg’s B5 medium, and nitrogen-free MS (MS–N) medium). All assemblies were highly complete (≥89% complete BUSCOs). Replicates clustered by species (median within-species r = 0.99 versus between-species r = 0.40; label-permutation *p* = 1 × 10^−4^), and species signatures selected on training replicates correctly assigned every held-out sample. GO and KEGG annotation composition differed among species, but no term reached a false discovery rate (FDR) ≤ 0.10 in species-specific enrichment tests. Medium effects were species-dependent: relative to basal MS, MS–N lowered protein and chlorophyll a and raised glucose and chlorophyll b in all species, and increased peroxidase activity in four of five species. Because the media differ in several components, these effects cannot be attributed to nitrogen alone. The five species have distinct, reproducible baseline transcriptomes and differ in their physiological responses to nutrient media, providing a hypothesis-generating resource for comparative duckweed research.

## 1. Introduction

Duckweeds are the smallest and most morphologically reduced flowering plants on Earth, comprising approximately 36 recognized species distributed across five genera, *Spirodela*, *Landoltia*, *Lemna*, *Wolffiella*, and *Wolffia*, within the monocot order Alismatales [1,2,3]. Despite their deceptively simple structure, consisting of a thallus-like frond lacking true stems or leaves and having highly variable root architecture, duckweeds are true vascular monocots capable of both rapid vegetative reproduction by frond budding and, under specific environmental conditions, sexual reproduction via flowering [4,5]. Under optimal conditions, their biomass doubling time is 24–48 h, placing them among the fastest-growing plants known [6,7]. The Lemnaceae display remarkable genomic diversity despite morphological conservatism. Genome sizes span a 13-fold range from approximately 150 Mb in *Spirodela polyrhiza* (*S. polyrhiza*) to over 1881 Mb in *Wolffia arrhiza*, yet all sequenced duckweed genomes encode a compact core proteome of approximately 19,000–22,400 protein-coding genes [5,8,9]. The sequencing of reference genomes for *S. polyrhiza* [5], *Lemna minor* (*L. minor*) [8], *Wolffia australiana* [10], and *Spirodela intermedia* [11], and a recent phylogenomic analysis spanning eleven Lemnaceae accessions [12], have collectively revealed ancient whole-genome duplications, widespread hybridization and polyploidization, and gene family contractions associated with the aquatic lifestyle and morphological reduction of the group.

Transcriptomic studies in individual duckweed species have provided important functional insights. RNA-seq analyses of *S. polyrhiza* under heat stress identified ERF and GRAS transcription factor networks in thermotolerance [13]. In *Lemna gibba* (*L. gibba*), integrated transcriptomic and metabolic flux analysis shown concurrent Calvin-Benson-Bassham cycle and oxidative pentose phosphate pathway activity, and demonstrated that nitrogen source profoundly reorganizes TCA cycle fluxes [14]. Post-transcriptional buffering between transcript and protein levels was documented during salicylic acid-induced flowering in *L. gibba* [15]. Photosynthetic adaptation to irradiance across *Lemna* species was shown to be species- and accession-specific [7], and single-nucleus RNA-seq of *Lemna minuta* revealed a simplified molecular cell-type atlas consistent with this species’ morphological reduction [16]. However, a systematic multi-species comparison of baseline transcriptomes across several Lemnaceae species grown under a common condition has been lacking, which limits our understanding of species-level molecular differences.

Beyond fundamental biology, duckweeds have attracted substantial biotechnological interest. Their high starch and protein content with protein concentrations and amino acid profiles comparable to soybean, combined with rapid growth on marginal water resources without soil requirements, positions them as sustainable feedstocks for biofuels, animal feed, and human nutrition [9,17,18]. Species of *Landoltia* and *Lemna* are explored for phytoremediation of municipal, agricultural, and industrial wastewater, achieving nitrogen and phosphorus removal efficiencies of up to 98% and 96%, respectively [19]. Duckweeds also accumulate diverse secondary metabolites including phenylpropanoids, polyamines, and flavonoids, mediated by rich complements of cytochrome P450 (CYP) enzymes [20,21]. Despite these features, the species-specific molecular programs underlying differential growth capacity and metabolite production are poorly resolved at the transcriptomic level.

Cross-species transcriptome comparisons have been used in other plant groups to distinguish shared from species-specific molecular responses [22,23]. Orthogroup-based comparative transcriptomic analysis, in which genes are clustered into evolutionarily related gene groups using tools such as OrthoFinder [24] provides a principled framework for cross-species expression comparison that accounts for variable gene copy numbers and paralog content arising from polyploidization. Functional annotation using Kyoto Encyclopedia of Genes and Genomes (KEGG) pathway analysis [25] and Gene Ontology (GO) term enrichment enables species-level differences in annotated gene content. This approach is particularly well-suited to the Lemnaceae, where polyploidy, hybridization, and gene family contractions create variable gene-to-orthogroup relationships across species [12]. To begin addressing this gap, we conducted a baseline comparative transcriptome analysis with an independent physiological analysis. First, we generated de novo transcriptome assemblies from five duckweed species, *L. minor*, *Lemna japonica* (*L. japonica*), *L. gibba*, *Landoltia punctata* (*La. punctata*), *and Lemna aequinoctialis* (*L. aequinoctialis*) grown under a single standard condition (basal Murashige and Skoog [MS] medium), and assessed their completeness with Benchmarking Universal Single-Copy Orthologs (BUSCO). Next, we compared orthogroup-level expression using principal component analysis (PCA), Pearson correlation matrices, and species signature scores. We then described GO and KEGG annotation composition and performed exploratory species-specific overrepresentation tests. Finally, in an independent experiment, we measured six physiological traits, including fresh weight biomass, protein concentration, glucose concentration, peroxidase activity, chlorophyll a concentration, and chlorophyll b concentration, of five duckweed species after 7 days of growth on four nutrient media. Together, these analyses can provide a comparative baseline resource for the molecular and physiological responses underlying duckweed diversity, and a set of testable hypotheses about species-level differences within the Lemnaceae. Because RNA-seq was performed only on basal MS, the transcriptomic data describe baseline differences among species and are not used to explain the medium responses.

## 2. Results

### 2.1. De Novo Transcriptome Assemblies Show High Completeness, and Deduplication Reduces Transcript Redundancy Across All Five Species

**Figure 1:**
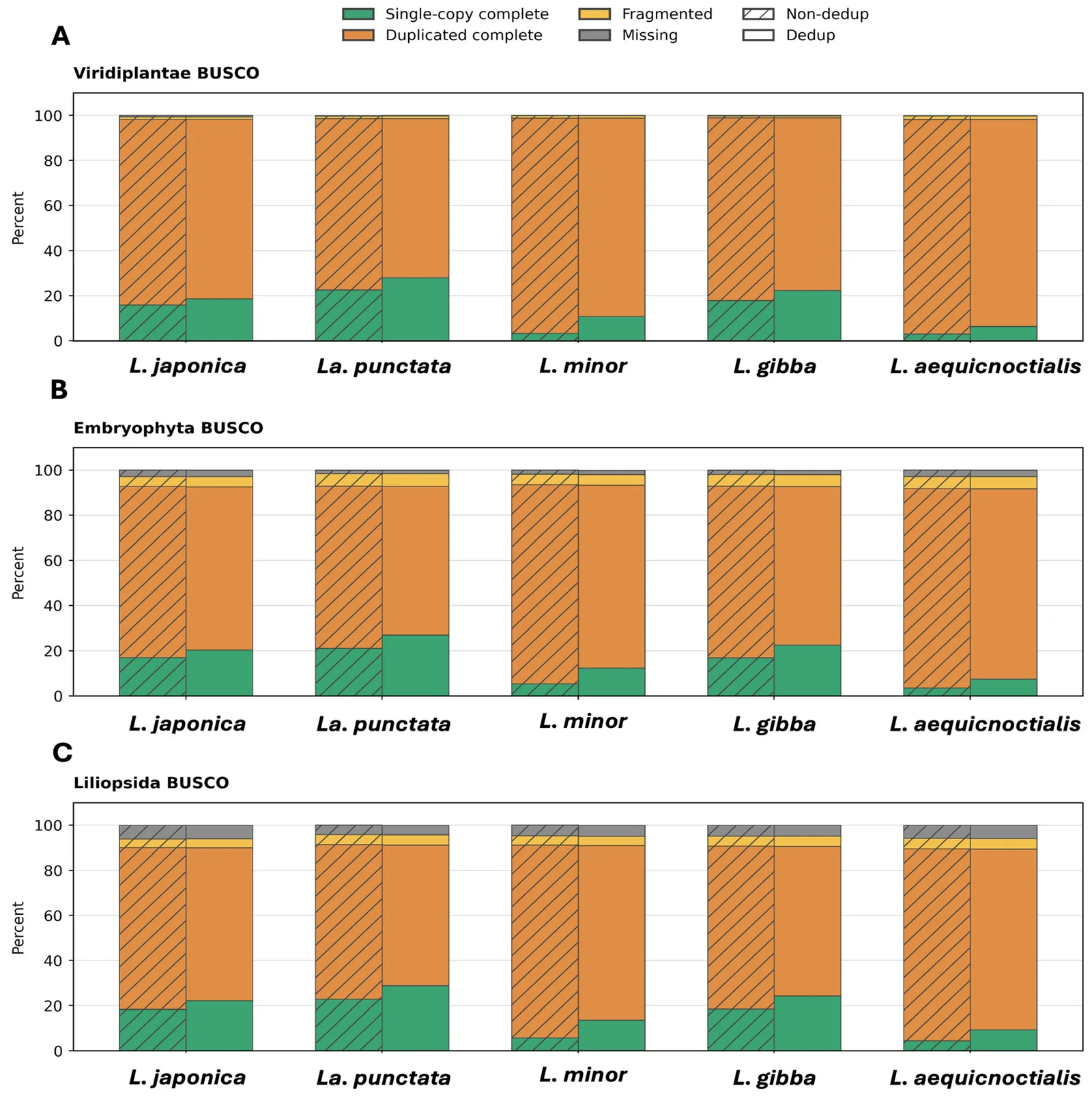
BUSCO performance assessment across five duckweed species (*L. japonica*, *La. punctata*, *L. minor*, *L. gibba*, *and L. aequinoctialis*) using three lineage datasets: (**A**) Viridiplantae, (**B**) Embryophyta, and (**C**) Liliopsida. For each species, paired bars show assembly metrics before deduplication (Non-dedup, hatched bars) and after deduplication (Dedup, solid bars). Stacked colors represent the percentage of single-copy complete (green), duplicated complete (orange), fragmented (yellow), and missing (grey) BUSCO genes. Abbreviations: BUSCO, Benchmarking Universal Single-Copy Orthologs; Dedup, deduplicated; Non-dedup, non-deduplicated.

We first assessed the quality and completeness of the de novo transcriptome assemblies generated from five duckweed species *L. minor*, *L. japonica*, *L. gibba*, *La. punctata*, *and L. aequinoctialis.* Non-deduplicated (non-dedup) and deduplicated (dedup) assemblies were evaluated with BUSCO [26,27] against three reference databases representing progressively more specific taxonomic levels: Viridiplantae (822 BUSCOs), Embryophyta (2026 BUSCOs), and Liliopsida (2821 BUSCOs). This approach yielded 30 assembly-database combinations that were evaluated in parallel (Figure 1, Table S1). Across the Viridiplantae BUSCO database (Figure 1A), all non-deduplicated assemblies reached high total BUSCO completeness, ranging from 98.2% to 98.9% across all species (Table S1). In all five species, complete BUSCOs were predominantly duplicated rather than single-copy, consistent with allelic, homeologous, paralogous, or isoform-level redundancy in the assembled transcripts. Following deduplication, the proportion of single-copy complete BUSCOs increased markedly across all species, while the proportion of duplicated BUSCOs decreased proportionally, with total completeness decreasing by 0–0.2 percentage points. The largest absolute gains in single-copy BUSCOs were observed for *La. punctata* and *L. japonica*. Nevertheless, duplicated BUSCOs remained the majority in all deduplicated assemblies, and BUSCO duplication alone cannot distinguish among allelic, homeologous, paralogous, and assembly-derived redundancy. The Embryophyta BUSCO database (Figure 1B) showed similar patterns across all five species. Non-deduplicated assemblies maintained high total completeness of 91.8–93.5% (Table S1), with a predominance of duplicated BUSCOs in all species. A small but visible fragmented BUSCO fraction was detected in several species, most notably in *La. punctata* and *L. aequinoctialis*. Deduplication again consistently converted duplicated BUSCO content to single-copy BUSCOs across all species, with decreases of 0.1–0.2 percentage points in total completeness. *La. punctata* and *L. japonica* continued to show the most substantial deduplication-driven improvement in single-copy BUSCO proportions under this database.

Under the Liliopsida BUSCO database (Figure 1C), which represents the most taxonomically specific reference set for monocots, non-deduplicated and deduplicated completeness values were 89.5–91.3% and 89.4–91.2%, respectively (Table S1), lower than those observed for the Viridiplantae and Embryophyta databases, with missing BUSCO fractions (grey) detectable across all species. Fragmented BUSCOs (yellow) ranged from 3.8% to 4.7% before deduplication and from 4.0% to 4.7% after deduplication, with the largest fractions in *L. aequinoctialis* and *L. gibba* (Table S1). The shift from duplicated to single-copy BUSCOs after deduplication was preserved consistently. *La. punctata* showed the lowest missing BUSCO fraction across the Liliopsida database (4.3%), followed by *L. minor* and *L. gibba* (both 4.8%), in both assembly versions (Table S1). Nonetheless, the BUSCO results indicate that all five transcriptome assemblies have high completeness and that deduplication reduces duplicated transcript representation. The original, non-deduplicated assemblies were used for downstream protein prediction and expression quantification; deduplicated assemblies were used for the paired BUSCO comparison.

### 2.2. Comparative Transcriptome Analysis Reveals Distinct Species-Specific Expression Profiles with High Within-Species Reproducibility

#### 2.2.1. Species Signatures Remain Specific When Evaluated on Held-Out Replicates

To characterize the global architecture of transcriptomic variation across five duckweed species grown on basal MS medium, we evaluated species signatures with a balanced internal validation scheme (Figures S1 and S2; Table S2) using all 243 balanced holdouts, each with two training samples and one held-out sample per species. Each of the 15 samples was withheld in 81 overlapping splits. The primary analysis selected 30 orthogroups (OGs) per signature from the 8040 orthogroups represented in all five species, with expression filtering, marker selection, and score calibration performed within each training set. In the primary configuration, each of the 15 samples had a positive cognate-minus-highest-non-cognate score margin in all 81 of its held-out appearances (Figure S1A,B). After first taking the median margin over these 81 appearances for each sample, the median across the 15 sample-level values was 1.9159 (first–third quartiles, 1.8676–2.1887). The minimum margin across all held-out records was 1.7098. Per-sample median margins were approximately 1.8–1.9 for *L. japonica*, *La. punctata*, and *L. minor*, 2.1–2.2 for *L. gibba*, and about 2.4 for *L. aequinoctialis*. These 1215 records arise from overlapping splits of 15 samples and are not independent observations. Positive margins were retained for every original sample in all its held-out appearances across the 30 configurations covering three orthogroup universes, two expression filters, and five marker counts (Figures S1C and S2A–C). At 30 markers and the two-training-sample threshold, the median of the 15 sample-level median margins was 2.2624 for all retained orthogroups, 1.9159 for shared orthogroups, and 1.8045 for the single-representative subset. Marker lists varied across splits. For the primary configuration, species-specific median Jaccard overlap between marker lists ranged from 0.8750 to 0.9355; 20–27 of the 30 markers per species were selected in every split (Figure S2D,E). Each split-specific shared-orthogroup list overlapped the original all-sample top 30 list by only 0–5 orthogroups, depending on species. This analysis evaluates the repeatability of a marker selection and scoring procedure within the existing expression matrix. Full balanced-holdout scores, marker selections, and sensitivity results are provided in Table S3.

#### 2.2.2. Principal Component Analysis (PCA) and Sample-to-Sample Correlation

**Figure 2:**
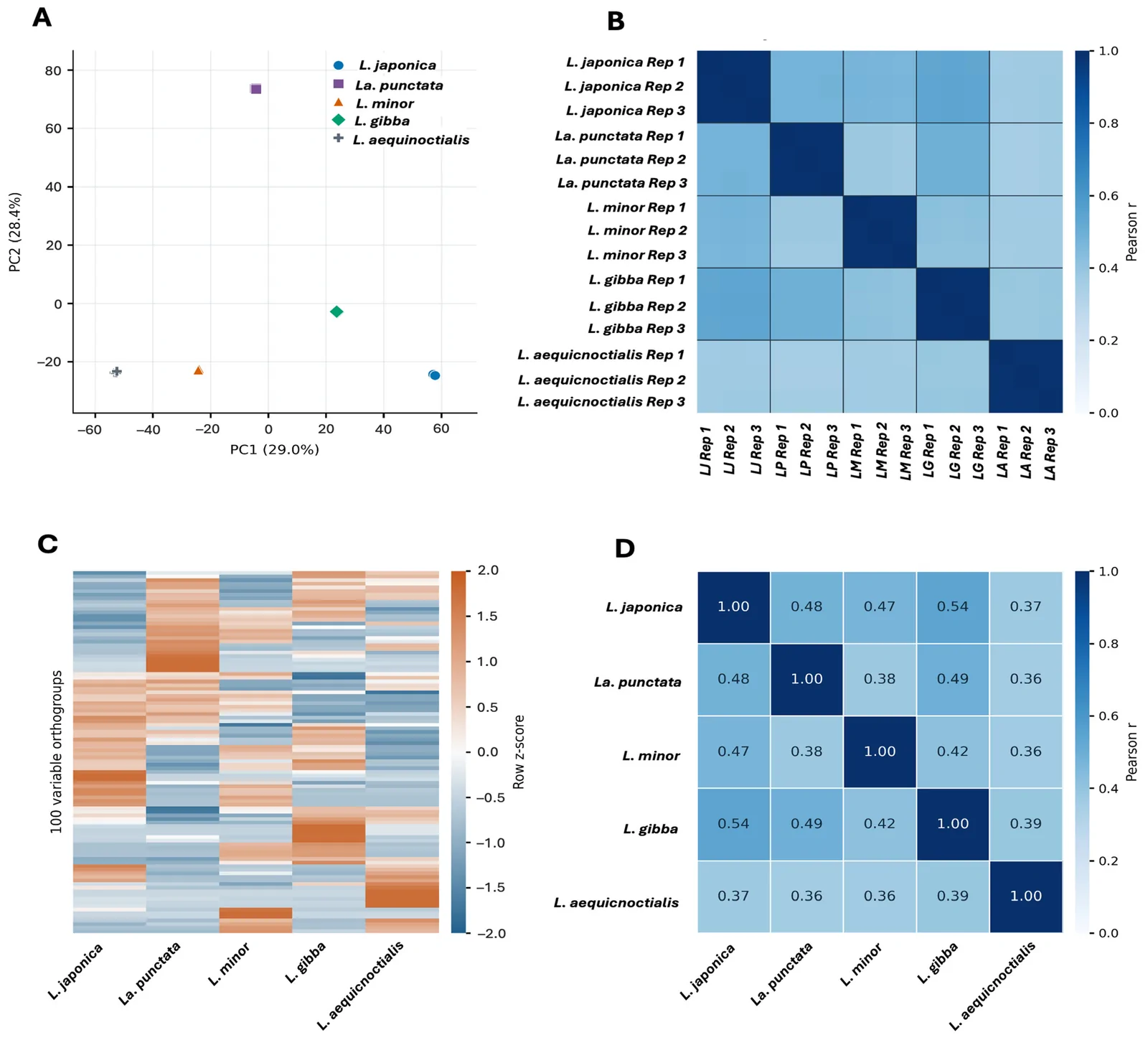
Orthogroup expression across five duckweed species (*L. minor*, *La. punctata*, *L. japonica*, *L. gibba*, *and L. aequinoctialis*) grown on basal MS medium. (**A**) Principal component analysis plot of the 5000 most variable orthogroups (log_2_(TPM + 1), centered and scaled to unit variance) for the 15 samples (*n* = 3 biological replicates per species); PC1 and PC2 explain 29.0% and 28.4% of the variance, respectively. (**B**) Sample-to-sample Pearson correlation matrix computed on log_2_(TPM + 1) values of 15,151 orthogroups with TPM ≥ 1 in at least 3 samples; median within-species r = 0.99 (15 sample pairs) and median between-species r = 0.40 (90 sample pairs). (**C**) Row z-scores of species-mean log_2_(TPM + 1) expression for the 100 orthogroups with the highest variance among species means; rows ordered by hierarchical clustering (Euclidean distance, average linkage). (**D**) Pearson correlation matrix of log_2_(mean TPM + 1) species profiles across the same 15,151 orthogroups (off-diagonal r = 0.36–0.54). TPM was averaged across replicates before log transformation. Heatmap color bars represent Pearson’s correlation coefficient (r, panels B and D) or standardized gene expression (z-score, panel C). Abbreviations: LA, *L. aequinoctialis*; LG, *L. gibba*; LJ, *L. japonica*; LM, *L. minor*; LP, *La. punctata*; OG, orthogroup; PC, principal component; PCA, principal component analysis; TPM, transcripts per million.

All transcriptomic comparisons in this section refer to plants grown on basal MS medium. PCA of the 5000 most variable orthogroups (log_2_(TPM + 1), standardized; Section 4.4) separated the 15 samples into five tight species clusters (Figure 2A). PC1 (29.0% of variance) separated *L. japonica* from *L. aequinoctialis* and *L. minor*, whereas PC2 (28.4%) separated *La. punctata* from the other species; together the first two components explained 57.4% of the variance, indicating that additional between-species structure is captured by higher components. Sample-to-sample Pearson correlation analysis across the 15,151 expressed orthogroups (Figure 2B; Table S4) showed the species grouping pattern observed in the signature score analysis. Pearson correlations of log_2_(TPM + 1) values had a median of 0.99 among the 15 within-species sample pairs (first–third quartiles, 0.9865–0.9912). The corresponding median among the 90 between-species sample pairs was 0.40 (0.3689–0.4746; Figure 2B and Figure S1D). The correlation heatmap revealed a clean 5 × 5 block structure along the diagonal, with high-correlation blocks for each species group. Because Pearson coefficients are descriptive and sample pairs that share a sample are not independent, we tested whether within-species correlations exceeded between-species correlations with a species-label permutation test that preserves this dependence structure. Within-species correlations were significantly higher than between-species correlations (*p* = 1.0 × 10^−4^, the smallest value attainable with 10,000 permutations). This result shows that, under the conditions tested, replicate-to-replicate variation is much smaller than the expression differences among species.

#### 2.2.3. The Top 100 Most Variable Orthogroups Presented Differences in Relative Expression Across Species

The 100 orthogroups with the greatest variance among the five log_2_-transformed species-mean TPM profiles were selected from the same filtered set used for correlations. Their row-standardised values show differences in relative expression among species (Figure 2C). Row z-scores ranged from −2.0 to +2.0 and were ordered by average-linkage hierarchical clustering using Euclidean distances between the five-species z-score profiles. Positive and negative z-scores indicate expression above and below an orthogroup’s mean across these five species. This heatmap is a descriptive display of selected variable orthogroups and does not test differential expression or regulatory suppression. Each species was represented by at least one such block, consistent with species-specific expression patterns under a common growth condition.

#### 2.2.4. Species-Level Pearson Correlation Matrix Describes Pairwise Expression Similarities

Pearson correlations between the five species-mean profiles ranged from 0.36 to 0.54 across the 10 unique pairs (median, 0.40; first–third quartiles, 0.37–0.48; Figure 2D). The highest correlation was between *L. japonica* and *L. gibba* (r = 0.54), followed by *La. punctata* and *L. gibba* (r = 0.49), *L. japonica* and *La. punctata* (r = 0.48), and the lowest was between *La. punctata* and *L. aequinoctialis* (0.36). *L. aequinoctialis* showed consistently lower correlations with other species (r = 0.36–0.39). These values describe similarities among log_2_-transformed species-mean TPM profiles. Cross-species correlations of de novo assembly-derived TPM values are also influenced by differences in assembly completeness, transcript redundancy, and orthogroup copy number; these coefficients should therefore be read as relative measures of expression-profile similarity rather than as evolutionary distances. The species-mean matrix is provided in Table S5.

### 2.3. GO Annotation Composition Differs Among Species, Whereas Species-Specific Overrepresentation Is Not Statistically Supported

#### 2.3.1. Normalized GO Composition Identifies Broad Functional Differences Across Species

**Figure 3:**
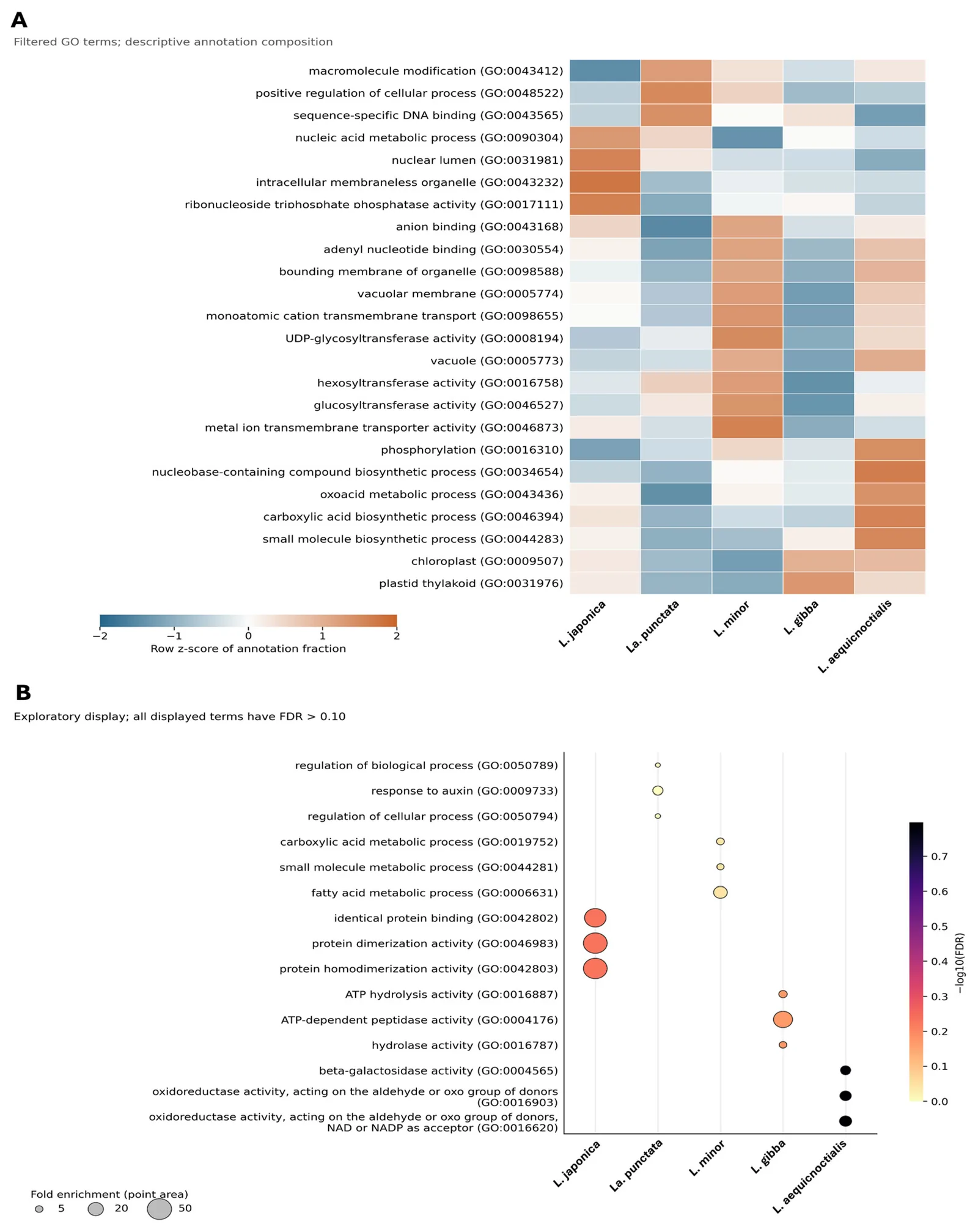
GO annotation composition and exploratory species-specific GO enrichment across five duckweed species (*L. minor*, *La. punctata*, *L. japonica*, *L. gibba*, *and L. aequinoctialis*). (**A**) GO annotation composition: for each species, the fraction of annotation-eligible orthogroups carrying each GO term was calculated (namespace-specific denominators); 24 terms were selected after removal of broad and redundant terms (Section 4.6), standardized by row z-score, and ordered by hierarchical clustering. Colors indicate relative representation across species and do not denote statistical enrichment or pathway activity. (**B**) Exploratory display of up to three top-ranked terms per species from one-sided hypergeometric tests of species-restricted orthogroups. Point area is proportional to max(fold enrichment, 1) (magnitude), and point color represents −log10(FDR) after Benjamini–Hochberg correction (statistical support). All displayed terms have FDR > 0.10, i.e., none are statistically significant at the pre-specified thresholds. Abbreviations: FDR, false discovery rate; GO, Gene Ontology.

To characterize differences in the functional composition of each species’ transcriptome, we calculated, for each species, the fraction of annotation-eligible orthogroups carrying each GO term and standardized these fractions across species as row z-scores (Figure 3A; Table S6). Examining the top GO terms, *L. japonica* showed relatively higher representation of nucleic acid metabolic process (GO:0090304), nuclear lumen (GO:0031981), intracellular membraneless organelle (GO:0043232), and ribonucleoside triphosphate phosphatase activity (GO:0017111), and lower representation of macromolecule modification (GO:0043412) and phosphorylation (GO:0016310). *La. punctata* showed relatively higher representation of macromolecule modification, positive regulation of cellular process (GO:0048522), and sequence-specific DNA binding (GO:0043565), and lower representation of anion binding (GO:0043168) and oxoacid metabolic process (GO:0043436). *L. minor* showed relatively higher representation of membrane- and transport-related terms, including bounding membrane of organelle (GO:0098588), vacuolar membrane (GO:0005774), vacuole (GO:0005773), monoatomic cation transmembrane transport (GO:0098655), and metal ion transmembrane transporter activity (GO:0046873), together with glycosyltransferase terms (UDP-glycosyltransferase activity, GO:0008194; hexosyltransferase activity, GO:0016758; glucosyltransferase activity, GO:0046527), and lower representation of nucleic acid metabolic process and chloroplast (GO:0009507). *L. gibba* showed relatively higher representation of chloroplast and plastid thylakoid (GO:0031976) terms and lower representation of glycosyltransferase and vacuolar/transport terms. *L. aequinoctialis* showed relatively higher representation of phosphorylation, nucleobase-containing compound biosynthetic process (GO:0034654), oxoacid metabolic process, carboxylic acid biosynthetic process (GO:0046394), and small molecule biosynthetic process (GO:0044283), and lower representation of sequence-specific DNA binding and nuclear lumen. These patterns describe the composition of annotated OGs. Row standardization expresses differences relative to the five-species mean and does not measure gene activation, repression or pathway activity.

#### 2.3.2. Species-Specific GO Overrepresentation Tests

We next tested whether GO terms were over-represented among orthogroups detected only in the focal species (species-restricted orthogroups) relative to all annotated orthogroups present in that species, using one-sided hypergeometric tests for terms with 5–500 background orthogroups and Benjamini–Hochberg correction within each species × annotation class (Table S7). Because these orthogroups were defined from assembled transcriptomes, species restriction reflects transcript detection rather than demonstrated genomic absence from the other species. No GO term with 5–500 background OGs was significantly overrepresented among species-restricted OGs at FDR ≤ 0.05 or ≤0.10 (Figure 3B). The term-level dot plot displays three ranked terms per species as an exploratory summary; all 15 displayed species–term results have FDR > 0.10. These included identical protein binding (GO:0042802), protein dimerization activity (GO:0046983), and protein homodimerization activity (GO:0042803) for *L. japonica*; response to auxin (GO:0009733) and regulation of biological and cellular processes (GO:0050789, GO:0050794) for *La. punctata;* fatty acid metabolic process (GO:0006631), carboxylic acid metabolic process (GO:0019752), and small molecule metabolic process (GO:0044281) for *L. minor*; ATP-dependent peptidase activity (GO:0004176), ATP hydrolysis activity (GO:0016887), and hydrolase activity (GO:0016787) for *L. gibba*; and beta-galactosidase activity (GO:0004565) and two oxidoreductase terms acting on aldehyde or oxo groups of donors (GO:0016903; GO:0016620, NAD or NADP as acceptor) for *L. aequinoctialis*. Large fold-enrichment values for some terms (e.g., up to approximately 50 for the *L. japonica* dimerization terms) reflect very small numbers of foreground orthogroups and are not accompanied by statistical support.

### 2.4. KEGG Annotation Composition and Species-Specific Overrepresentation Tests

#### 2.4.1. KEGG Pathway Composition Describes Annotation-Level Differences

**Figure 4:**
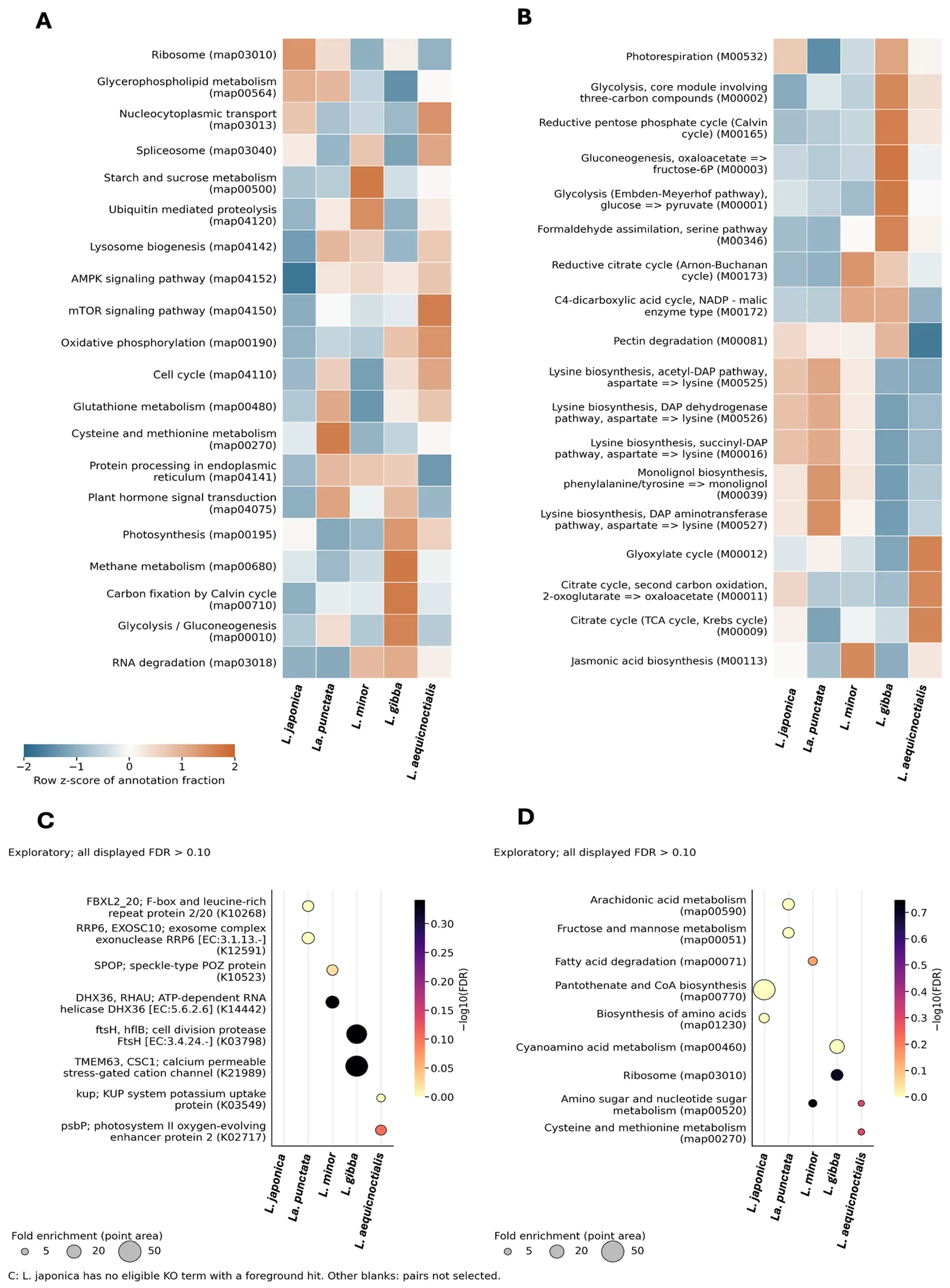
KEGG annotation composition and exploratory species-specific KEGG enrichment across five duckweed species. (**A**) KEGG pathway composition (20 most variable pathways) and (**B**) KEGG module composition (18 most variable modules), shown as row z-scores of the fractions of annotation-eligible orthogroups carrying each term. Colors indicate relative representation of annotated genes across species and do not denote statistical enrichment, pathway activation, or metabolic flux. (**C**) Exploratory display of the top-ranked KEGG Orthology (KO) terms and (**D**) KEGG pathway terms from one-sided hypergeometric tests of species-restricted orthogroups (up to two per species). Point area is proportional to max(fold enrichment, 1), and point color represents −log10(FDR) after Benjamini–Hochberg correction. All displayed terms have FDR > 0.10. In panel (**C**), *L. japonica* had no eligible KO term with a foreground hit; other blank positions indicate species–term pairs not selected for display. Abbreviations: KEGG, Kyoto Encyclopedia of Genes and Genomes; KO, KEGG Orthology.

KEGG pathway composition was summarized with the same framework (fractions of annotation-eligible orthogroups followed by row z-scores; Figure 4A; Table S6); global and overview maps and maps describing human-disease, drug-development, or animal-specific contexts were omitted from the display, and the 20 most variable pathways are shown. As for GO, these values describe the relative representation of annotated genes and do not indicate pathway activation, enzyme activity, or metabolic flux. *L. gibba* showed relatively higher fractions of carbon fixation by the Calvin cycle (map00710), glycolysis/gluconeogenesis (map00010), photosynthesis (map00195), and methane metabolism (map00680), and a lower fraction of glycerophospholipid metabolism (map00564). *La. punctata* had relatively higher fractions of cysteine and methionine metabolism (map00270), glutathione metabolism (map00480), plant hormone signal transduction (map04075), and protein processing in the endoplasmic reticulum (map04141), and lower representation of photosynthesis, methane metabolism, and RNA degradation (map03018). *L. minor* showed relatively higher fractions of starch and sucrose metabolism (map00500) and ubiquitin-mediated proteolysis (map04120), and lower representation of glutathione metabolism and cell cycle (map04110). *L. japonica* showed relatively higher fractions of ribosome (map03010) and glycerophospholipid metabolism, and the lowest representation of AMPK signaling (map04152). *L. aequinoctialis* showed relatively higher fractions of mTOR signaling (map04150), oxidative phosphorylation (map00190), nucleocytoplasmic transport (map03013), spliceosome (map03040), and cell cycle, and lower representation of protein processing in the endoplasmic reticulum. Because the AMPK and mTOR maps are defined primarily from non-plant systems, their plant annotations correspond to conserved components (e.g., SnRK1- and TOR-associated orthologs) rather than to complete signaling pathways.

#### 2.4.2. KEGG Module Composition Describes Annotation-Level Differences

KEGG module composition (18 most variable modules; Figure 4B; Table S9) was broadly consistent with the pathway-level patterns. *L. gibba* showed relatively higher representation of photorespiration (M00532), the glycolysis core and full glycolysis modules (M00002, M00001), gluconeogenesis (M00003), the reductive pentose phosphate (Calvin) cycle (M00165), and the serine pathway of formaldehyde assimilation (M00346), and lower representation of the lysine biosynthesis and monolignol biosynthesis (M00039) modules. *La. punctata* showed relatively higher representation of four lysine biosynthesis modules (M00016, M00525, M00526, M00527) and monolignol biosynthesis (M00039), and lower representation of photorespiration and the citrate cycle (M00009). *L. minor* showed relatively higher representation of the C4-dicarboxylic acid cycle, NADP-malic enzyme type (M00172), the reductive citrate cycle (M00173), and jasmonic acid biosynthesis (M00113). *L. aequinoctialis* showed relatively higher representation of the glyoxylate cycle (M00012) and citrate cycle modules (M00009, M00011), and the lowest representation of pectin degradation (M00081). *L. japonica* showed no strongly elevated modules and the lowest representation of the glycolysis core module (M00002). Several modules (e.g., M00173 and M00346) are defined from prokaryotic pathways; their representation in duckweed reflects enzymes shared with plant central and one-carbon metabolism rather than the presence of these complete pathways.

#### 2.4.3. Species-Specific KO and Pathway Enrichment

Species-specific KO and pathway enrichment tests (species-specific foreground, as for GO; Figure 4C,D; Table S10) did not identify any term at FDR ≤ 0.10. For KO terms, the largest −log10(FDR) value was approximately 0.33 (FDR ≈ 0.47); for pathway terms, it was approximately 0.75 (FDR ≈ 0.18). No KO term with at least one foreground orthogroup was eligible for testing in *L. japonica*. The top-ranked terms are displayed as an exploratory summary and are described here using their KEGG definitions: K10268 (FBXL2_20; F-box and leucine-rich repeat protein 2/20) and K12591 (RRP6/EXOSC10; exosome complex exonuclease RRP6) for *La. punctata*; K10523 (SPOP; speckle-type POZ protein) and K14442 (DHX36/RHAU; ATP-dependent RNA helicase DHX36) for *L. minor*; K03798 (ftsH/hflB; ATP-dependent zinc metalloprotease FtsH) and K21989 (TMEM63/CSC1; calcium-permeable stress-gated cation channel) for *L. gibba*; and K03549 (kup; KUP-type potassium uptake protein) and K02717 (psbP; photosystem II oxygen-evolving enhancer protein 2) for *L. aequinoctialis*. At the pathway level, the top-ranked terms were pantothenate and CoA biosynthesis (map00770) and biosynthesis of amino acids (map01230) for *L. japonica*; arachidonic acid metabolism (map00590) and fructose and mannose metabolism (map00051) for *La. punctata*; fatty acid degradation (map00071) and amino sugar and nucleotide sugar metabolism (map00520) for *L. minor*; cyanoamino acid metabolism (map00460) and ribosome (map03010) for *L. gibba*; and amino sugar and nucleotide sugar metabolism (map00520) and cysteine and methionine metabolism (map00270) for *L. aequinoctialis*.

### 2.5. Physiological Profiling Across Five Species and Four Nutrient Media

To evaluate the physiological differences, we measured six key physiological parameters: fresh weight biomass, protein concentration, glucose concentration, peroxidase activity, chlorophyll a concentration, and chlorophyll b concentration in all five duckweed species (*L. minor*, *L. japonica*, *L. gibba*, *La. punctata*, and *L. aequinoctialis*) grown under four distinct nutrient media: Basal MS, Hoagland salt solution, B5 medium, and nitrogen-free MS (MS–N). Each species × medium combination comprised three independent biological replicates, each measured in three technical replicates that were averaged before analysis. The media differ not only in nitrogen supply but also in the concentrations of potassium, phosphate, calcium, magnesium, iron, micronutrients, and organic supplements (Table 1). Effects are therefore described as medium effects; only the comparison between basal MS and MS–N approximates a nitrogen contrast, and even this comparison also involves a 16-fold lower potassium concentration and the absence of vitamins in MS–N (Table 1). Two-way ANOVA results (species, medium, and species × medium interaction) are summarized in Table 2.

#### 2.5.1. Change in Biomass Accumulation of Different Species-Specific Under Standard Conditions

**Figure 5:**
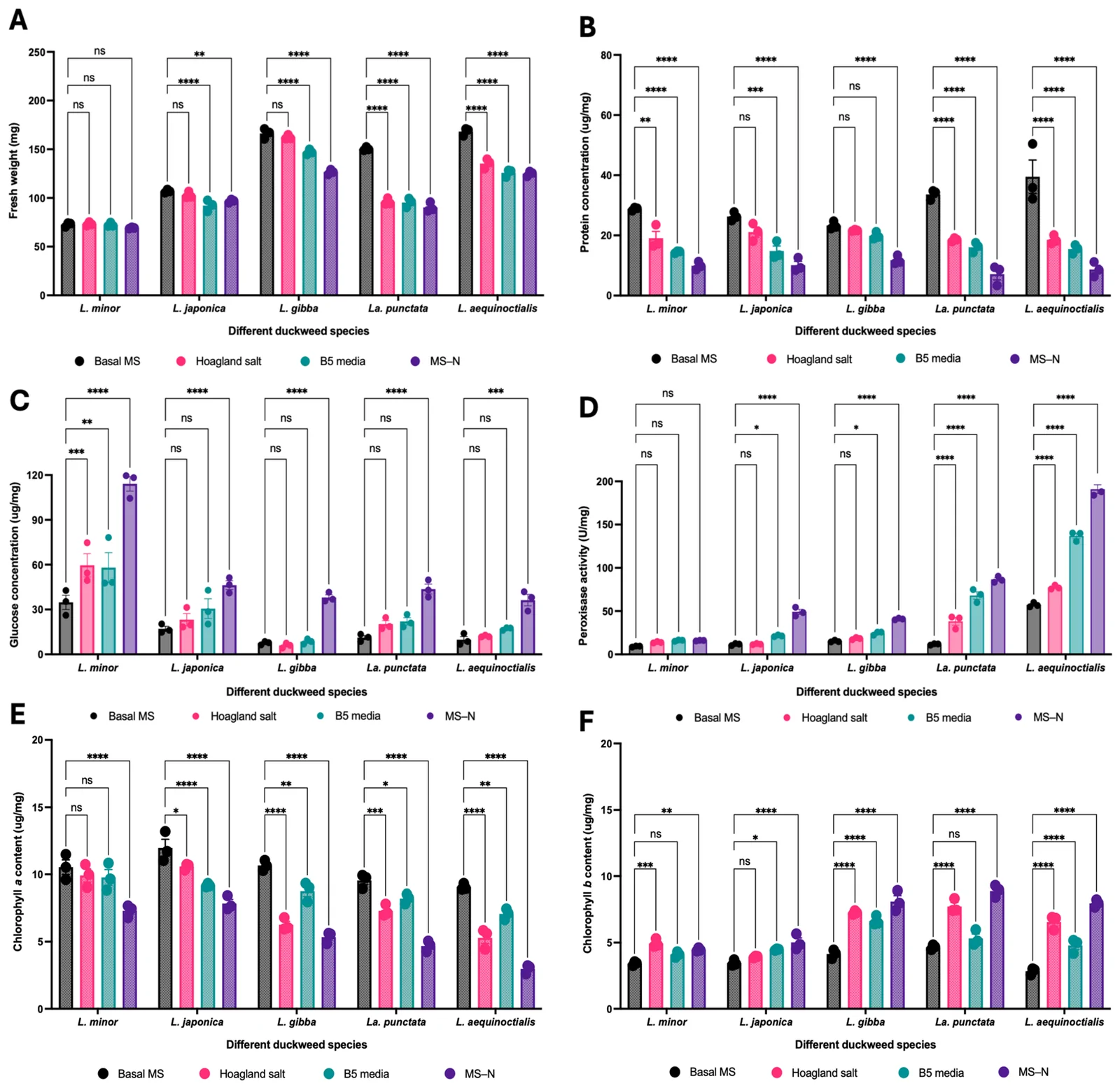
Physiological, biochemical, and photosynthetic responses of five duckweed species after 7 days of cultivation under four growth media (Basal MS, Hoagland salt, B5 media, and nitrogen-free MS, MS–N). (**A**) Biomass response measured by fresh weight (mg). (**B**) Protein concentration (μg mg^−1^ FW). (**C**) Glucose concentration (μg mg^−1^ FW). (**D**) Peroxidase activity (U mg^−1^ FW). (**E**) Chlorophyll a concentration (μg mg^−1^ FW). (**F**) Chlorophyll b concentration (μg mg^−1^ FW). Bars show means ± SE of three biological replicates (*n* = 3); each point is one biological replicate, calculated as the mean of three technical replicates. Data were analyzed by two-way ANOVA (species × medium) followed by Tukey’s multiple comparisons test; for clarity, only comparisons of each medium with basal MS within each species are shown (* *p* < 0.05, ** *p* < 0.01, *** *p* < 0.001, **** *p* < 0.0001; ns, non-significant). ANOVA statistics are given in Table 2. Abbreviations: FW, fresh weight; MS, Murashige and Skoog medium; MS–N, nitrogen-free MS medium.

Fresh weight was affected by species, medium, and their interaction (all *p* < 0.0001; Table 2; Figure 5A). On basal MS, *L. aequinoctialis* (168.2 mg) and *L. gibba* (166.2 mg) produced the highest fresh weight and did not differ from each other, followed by *La. punctata* (150.4 mg) and *L. japonica* (106.9 mg), whereas *L. minor* produced the least (72.7 mg); all other between-species differences on basal MS were significant (*p* < 0.0001). *L. minor* showed no significant difference in fresh weight between basal MS and any other medium (69.2–73.2 mg; *p* ≥ 0.59). In *L. japonica*, fresh weight did not differ between basal MS and Hoagland medium but was lower on B5 (92.2 mg; *p* < 0.0001) and MS–N (96.8 mg; *p* = 0.004). In *L. gibba*, fresh weight did not differ between basal MS and Hoagland medium but was lower on B5 (147.1 mg) and MS–N (126.6 mg; both *p* < 0.0001). *La. punctata* and *L. aequinoctialis* showed significant reductions on all three alternative media relative to basal MS (all *p* < 0.0001); on MS–N, fresh weights were 90.3 and 125.0 mg, respectively. Because Hoagland and B5 media caused reductions in these two species similar in size to those on MS–N, the decreases cannot be attributed specifically to nitrogen supply. Fresh weight was measured at a single 7-day endpoint and was not normalized to initial biomass, so these values reflect both growth and species differences in frond size.

#### 2.5.2. Change in Protein Concentration Across Five Duckweed Species

Protein concentration (μg mg^−1^ fresh weight [FW]) was strongly affected by medium (*p* < 0.0001; η*^2^_p_* = 0.91), and the medium effect depended on species (species × medium interaction, *p* < 0.0001), whereas the main effect of species was not significant (*p* = 0.226; Table 2; Figure 5B). On basal MS, mean values ranged from 23.28 μg mg^−1^ FW in *L. gibba* to 39.49 μg mg^−1^ FW in *L. aequinoctialis*, with *La. punctata* (33.61), *L. minor* (28.82), and *L. japonica* (26.28) intermediate; *L. aequinoctialis* exceeded *L. minor*, *L. japonica*, and L. gibba (*p* ≤ 0.0005), and *La. punctata* exceeded *L. japonica* (*p* = 0.027) and *L. gibba* (*p* = 0.0007). MS–N produced the lowest protein concentration in every species, with significant reductions relative to basal MS in all five species (all *p* < 0.0001), corresponding to decreases of approximately 49–79%. Responses to Hoagland and B5 media were species-dependent: in *L. minor*, protein was lower on Hoagland (*p* = 0.001) and B5 (*p* < 0.0001); in *L. japonica*, it did not differ on Hoagland but was lower on B5 (*p* = 0.0001); in *L. gibba*, neither Hoagland nor B5 differed from basal MS; and in *La. punctata* and *L. aequinoctialis*, protein was lower on both media (*p* < 0.0001). The largest proportional decreases on MS–N occurred in *La. punctata* (79%; 7.13 μg mg^−1^) and *L. aequinoctialis* (78%; 8.65 μg mg^−1^). Because the species ranking on basal MS was largely lost on the alternative media, the species effect averaged across media was not significant.

#### 2.5.3. Change in Glucose Concentration Across Five Duckweed Species

Glucose concentration (μg mg^−1^ FW), measured at a single endpoint, was affected by species, medium, and their interaction (all *p* < 0.0001; Table 2) and increased on MS–N relative to basal MS in all five species (Figure 5C). On basal MS, *L. minor* had a higher glucose concentration (34.77 μg mg^−1^) than each of the other species (7.57–17.06 μg mg^−1^; *p* ≤ 0.029). In *L. minor*, the average glucose concentration was also higher on Hoagland (59.50 μg mg^−1^; *p* = 0.0006), B5 (58.04 μg mg^−1^; *p* = 0.0013), and MS–N (114.1 μg mg^−1^; *p* < 0.0001). The approximately 3.3-fold increase in *L. minor* on MS–N produced the highest glucose concentration observed across all species and media. In *L. japonica*, glucose did not differ between basal MS and Hoagland or B5 media but was higher on MS–N (46.35 μg mg^−1^; *p* < 0.0001). In *L. gibba*, no significant differences were detected between Basal MS, Hoagland salt, and B5 media, but a highly significant glucose increase was observed under MS–N (38.07 μg mg^−1^; *p* < 0.0001). *La. punctata* showed a modest but statistically significant increase in glucose under MS–N (43.70 μg mg^−1^; *p* < 0.0001) relative to Basal MS, with no significant differences between other media pairs. *L. aequinoctialis* showed no significant difference between basal MS and Hoagland or B5 media, but glucose was higher on MS–N (36.31 μg mg^−1^; *p* = 0.0002). Relative to basal MS, these increases corresponded to approximately 2.7-fold (*L. japonica*) to 5.0-fold (*L. gibba*). Thus, the glucose response to MS–N was consistent across species, whereas responses to Hoagland and B5 media were detected only in *L. minor*.

#### 2.5.4. Change in Peroxidase Activity Under Different Media Composition

Guaiacol peroxidase activity (U mg^−1^ FW) differed markedly among species in both its basal level and its responsiveness to medium (Figure 5D), with significant effects of species, medium, and their interaction (all *p* < 0.0001; Table 2). On basal MS, *L. aequinoctialis* had higher activity (57.16 U mg^−1^) than every other species (all *p* < 0.0001), whereas the other four species did not differ from one another (9.13–15.18 U mg^−1^). In *L. minor*, activity did not differ significantly between basal MS and any other medium (9.13, 13.91, 16.05, and 16.03 U mg^−1^ on basal MS, Hoagland, B5, and MS–N, respectively; *p* ≥ 0.13). In *L. japonica* and *L. gibba*, activity did not differ on Hoagland medium but was higher on B5 (21.42 U mg^−1^, *p* = 0.014, and 24.57 U mg^−1^, *p* = 0.022, respectively) and on MS–N (49.05 and 40.89 U mg^−1^; both *p* < 0.0001). In *La. punctata*, activity increased on all three alternative media (all *p* < 0.0001), from 11.66 U mg^−1^ on basal MS to 38.21 (Hoagland), 68.05 (B5), and 86.51 U mg^−1^ (MS–N), the largest proportional increase observed (≈7.4-fold). *L. aequinoctialis* also showed increases on all alternative media (all *p* < 0.0001), reaching 77.41 (Hoagland), 136.9 (B5), and 190.8 U mg^−1^ (MS–N), the highest values in the dataset (approximately 3.3-fold over basal MS). On MS–N, *L. aequinoctialis* activity exceeded that of all other species (all *p* < 0.0001). Because guaiacol peroxidase activity was the only redox-related parameter measured, these data show changes in enzyme activity under different media but do not establish ROS accumulation, oxidative damage, or ROS-scavenging capacity.

#### 2.5.5. Changes in Chlorophyll a Concentration Across All Five Species

Chlorophyll a concentration (μg mg^−1^ FW) was affected by species, medium, and their interaction (all *p* < 0.0001; Table 2; Figure 5E). On basal MS, values ranged from 9.07 (*L. aequinoctialis*) to 11.97 (*L. japonica*); *L. japonica* exceeded *L. minor* (*p* = 0.040), *La. punctata* (*p* = 0.0001), and *L. aequinoctialis* (*p* < 0.0001). MS–N produced the lowest chlorophyll a concentration in all five species (all *p* < 0.0001 versus basal MS), with values of 7.31 (*L. minor*), 7.85 (*L. japonica*), 5.35 (*L. gibba*), 4.69 (*La. punctata*), and 2.98 (*L. aequinoctialis*), corresponding to decreases of approximately 31–67%. Responses to Hoagland and B5 media varied: *L. minor* showed no difference on either medium, whereas the other four species showed significant reductions on both (*p* = 0.042 to *p* < 0.0001), and *L. gibba* and *L. aequinoctialis* showed particularly large reductions on Hoagland medium (to 6.27 and 5.29, respectively; both *p* < 0.0001). The consistent decrease on MS–N is consistent with the nitrogen requirement of chlorophyll biosynthesis [28], although other nutritional differences among media may also contribute.

#### 2.5.6. Changes in Chlorophyll b Concentration Across All Five Species and Different Media

Chlorophyll b concentration (μg mg^−1^ FW) responded differently from chlorophyll a, with significant effects of species, medium, and their interaction (all *p* < 0.0001; Table 2; Figure 5F). Chlorophyll b increased on MS–N in all five species. On basal MS, values ranged from 2.84 (*L. aequinoctialis*) to 4.66 (*La. punctata*). MS–N increased chlorophyll b relative to basal MS in all five species (*L. minor*, *p* = 0.0096; all others, *p* < 0.0001), with the largest increases in *L. gibba* (8.11), *La. punctata* (8.91), and *L. aequinoctialis* (7.96). Hoagland medium increased chlorophyll b in *L. minor* (*p* = 0.0001), *L. gibba*, *La. punctata*, and *L. aequinoctialis* (all *p* < 0.0001) but not in *L. japonica*, and B5 medium increased it in *L. japonica* (*p* = 0.013), *L. gibba*, and *L. aequinoctialis* (both *p* < 0.0001) but not in *L. minor* or *La. punctata*. As a result of the opposite changes in chlorophyll a and b, the chlorophyll a/b ratio calculated from group means decreased from 2.0–3.5 on basal MS to 0.4–1.6 on MS–N.

## 3. Discussion

All five duckweed transcriptome assemblies achieved high BUSCO scores (98.2–98.9% Viridiplantae, 91.8–93.5% Embryophyta, 89.4–91.3% Liliopsida), indicating assembly quality sufficient for robust orthogroup-level comparative analysis (Figure 1) [26,27,29]. Deduplication at 99% nucleotide identity shifted part of the duplicated BUSCO content to single-copy BUSCOs without reducing total completeness, but duplicated BUSCOs remained the majority in all assemblies. This is expected when homeologous or paralogous copies differ by more than 1% at the nucleotide level or when alternative isoforms are retained, and it is compatible with the hybridization and polyploidization documented in the Lemnaceae [5,12]. The larger gains in *La. punctata* and *L. japonica* may indicate greater collapsible redundancy in these assemblies, but BUSCO duplication cannot distinguish allelic, homeologous, paralogous, and assembly-derived copies. Because deduplication was evaluated as an assembly quality comparison, expression was quantified against the non-deduplicated assemblies and summarized from one representative transcript per Trinity gene; this aggregation reduces, but does not remove, the influence of redundancy and isoform structure on cross-species comparisons. Fragmented BUSCOs in *L. japonica* and *L. aequinoctialis* under Embryophyta/Liliopsida may reflect sequence divergence at monocot-specific loci, incomplete transcript reconstruction, or low expression of the corresponding genes under the sampled condition; distinguishing these possibilities will require reference genomes for these species [12]. Replicates were highly concordant (median within-species r = 0.99), whereas between-species correlations were much lower (median r = 0.40; Figure 2A,B), and this difference was supported by a label-permutation test that accounts for the dependence among sample pairs (Figure S1D). The species signatures also correctly assigned all held-out replicates across 243 balanced splits and were robust to signature size, filtering threshold, and candidate set (Figures S1 and S2). These analyses address the circularity of selecting and evaluating signatures on the same samples. However, they remain internal to one experiment with one accession per species, and species identity may be partly confounded with culture batch. Because duckweed physiology can vary among accessions of the same species [7], we still need to test the extent to which these signatures represent species rather than accession characteristics. Species-mean correlations ranged from 0.36 to 0.54 (Figure 2D). *L. aequinoctialis* was the least similar to the other four species, which is consistent with its placement separating from *L. minor*, *L. gibba*, and *L. japonica* [1]. However, expression similarity did not simply mirror taxonomy: the intergeneric pairs involving *La. punctata* were not the least similar, and *L. japonica*, which has been described as a hybrid between *L. minor* and *L. turionifera* [12,30,31], was more similar to *L. gibba* than to *L. minor*. Expression-profile similarity therefore appears to combine phylogenetic relatedness with lineage-specific regulatory and technical factors, including assembly redundancy and orthogroup composition. Formal tests of whether expression divergence tracks phylogeny or ecology will require more species and accessions analyzed within a phylogenetic framework [12].

The functional analyses distinguish annotation composition from statistical overrepresentation. GO and KEGG annotation composition differed among species (Figure 3A and Figure 4A,B). Still, these differences describe the relative representation of annotated genes in each transcriptome and do not measure pathway activity, enzyme activity, metabolite levels, or metabolic flux. Moreover, none of the species-specific GO, KO, or pathway enrichment tests reached the pre-specified FDR ≤ 0.10 threshold (Figure 3B and Figure 4C,D). The lack of statistical support may reflect the small numbers of species-specific orthogroups, which depend on transcript detection in each assembly, incomplete annotation of de novo assembled transcripts, annotation transfer from distantly related species [32], and the multiple-testing burden across many terms, which are recognized limitations of annotation-based enrichment analyses [33,34]. We therefore treat the annotation patterns as hypotheses rather than conclusions. Some patterns were consistent across annotation levels and may merit targeted follow-up. In *L. gibba*, relatively higher representation of plastid-related GO terms coincided with higher representation of Calvin cycle, glycolysis/gluconeogenesis, and photorespiration annotations at the pathway and module levels. This pattern is of interest given previous transcriptomic and flux analyses of central carbon metabolism in *L. gibba* [14], but whether it corresponds to differences in photosynthetic capacity would need to be tested with gas-exchange, enzyme activity, or flux measurements. In *La. punctata*, relatively higher representation of cysteine and methionine metabolism, glutathione metabolism, lysine biosynthesis, and monolignol biosynthesis annotations suggests hypotheses about sulfur amino acid, thiol, and phenylpropanoid metabolism that could be examined by metabolite profiling [20,21,35]. In *L. minor*, the relative over-representation of vacuolar membrane, cation transport, glycosyltransferase, and starch and sucrose metabolism annotations suggests possible differences in transport- and glycosylation-related gene content. In *L. aequinoctialis*, citrate cycle and glyoxylate cycle module representation and biosynthetic process terms suggest differences in central metabolism-related gene content. Each of these hypotheses requires validation at the transcript level, protein level, and metabolite level, particularly because transcript and protein abundances can be decoupled in duckweed and in other systems [15,36]. Although both the RNA-seq and the physiological experiments included basal MS, they were performed independently; we therefore interpret only the comparative transcriptomic profiles across the five duckweed species under basal controlled growth.

All five species responded to changes in nutrient medium, but the magnitude, and in some cases the direction, of the responses differed among species. Relative to basal MS, MS–N lowered protein and chlorophyll a and raised glucose and chlorophyll b in all five species, and lowered fresh weight and increased peroxidase activity in four species. These patterns are broadly consistent with the well-documented effects of nitrogen limitation, including reduced protein synthesis and accumulation of carbohydrates when nitrogen is limiting [37]; stress-associated carbohydrate (starch) accumulation has also been reported in *L. aequinoctialis* [38]. However, the media differ in several components besides nitrogen (Table 1). Basal MS contains approximately 60 mM nitrogen, B5 approximately 27 mM, and Hoagland’s No. 2 formulation approximately 15 mM, but these media also differ in their ammonium-to-nitrate ratio, potassium, phosphate, calcium, magnesium, iron chelate, micronutrient, and vitamin content. Because the nitrogen-free formulation omits KNO_3_ without a compensating potassium salt, MS–N also contains 16-fold less potassium than basal MS (1.25 versus 20.0 mM) and, unlike basal MS, contains no vitamins (Table 1). Accordingly, responses to Hoagland and B5 media cannot be ranked along a single nutrient gradient, and even the MS–N response should be interpreted as a response to nitrogen omission together with any accompanying changes in medium composition.

Species differences in responsiveness were nonetheless evident: the species × medium interaction was significant for all six traits (Table 2). *L. minor* showed no significant change in fresh weight or peroxidase activity on any medium, but showed the largest absolute increase in glucose concentration on MS–N and was the only species in which glucose also increased on Hoagland and B5 media. *La. punctata* and *L. aequinoctialis* showed the largest proportional decreases in protein concentration on MS–N, and both showed reductions in fresh weight on all alternative media. *L. aequinoctialis* combined the highest basal peroxidase activity with the largest absolute increases on alternative media, whereas *La. punctata* showed the largest proportional increase. Because the assays were performed after only 7 days, these differences may partly reflect species differences in growth rate, frond size, and internal nitrogen reserves rather than differences in long-term nutrient tolerance; duckweed species are known to differ widely in growth strategies and stress survival [39]. Protein was measured with the Bradford assay, which quantifies dye-binding soluble protein, and all traits were normalized to fresh weight, which can vary with tissue water content; dry-weight-based measurements would be needed to compare these values directly with reported duckweed protein contents, which are typically expressed on a dry-weight basis [17].

Peroxidase activity increased on alternative media in four of five species, and, at the treatment level, the media with the highest peroxidase activity generally had the lowest chlorophyll a concentration. Increases in class III peroxidase activity are commonly reported under abiotic stress, including in *L. aequinoctialis* [40,41,42], but class III peroxidases also participate in cell-wall formation, lignification, and auxin metabolism [43]. However, peroxidase activity alone is not a measure of ROS accumulation, oxidative damage, or ROS-scavenging capacity, and robust assessment of oxidative stress requires combined measurements of oxidants, oxidative damage markers, and multiple antioxidant components [44]; we did not measure H_2_O_2_, lipid peroxidation, or other antioxidant enzymes. We therefore describe the peroxidase data as a medium-dependent change in enzyme activity and do not infer ROS management from them. Likewise, with three biological replicates per group, we did not test correlations between peroxidase activity and chlorophyll content, and any such association would not imply a causal relationship. The decrease in chlorophyll a on MS–N is consistent with the nitrogen requirement of chlorophyll biosynthesis [28]. The concurrent increase in chlorophyll b, leading to calculated chlorophyll a/b ratios below 1 in three species, is less straightforward. A lower a/b ratio is usually associated with a relatively larger light-harvesting antenna [45], but ratios below 1 are unusual in green tissues, in which values of approximately 2.5–3.5 are typical [46]. Because chlorophyll was estimated spectrophotometrically from crude 80% acetone extracts, spectral contributions from chlorophyll degradation products formed under nutrient limitation [47] or from other pigments may affect the estimates, particularly when chlorophyll a is low. We therefore do not interpret the chlorophyll b increase as evidence of antenna remodeling; confirmation by HPLC-based pigment analysis and measurement of light-harvesting complex proteins would be required.

However, there are a few limitations of our study that should be noted. Our transcriptomic and physiological profiling used a single standardized condition set (Basal MS for transcriptomics; four media for physiology), so condition-dependent transcriptomic divergence across various parameters remains uncharacterized. The transcriptomic and physiological experiments were not matched, so transcriptomic signatures cannot be linked to the medium responses. Each species was represented by a single accession and three biological replicates, which limits generalization and statistical power. The physiological assays covered a short (7-day) period, used fresh-weight normalization, and compared media that differ in several nutrients besides nitrogen, including lower potassium and the absence of vitamins in MS–N. Also, bulk RNA-seq cannot resolve cell-type-specific contributions; given the Lemnaceae’s epidermal, mesophyll, vascular, and meristematic cell types, single-nucleus RNA-seq, as applied to *Lemna minuta* [16], would clarify whether species differences reflect cell-type composition, within-cell-type regulation, or both. Also, no direct measurements of metabolic flux, metabolite pools, or ROS were made, were not validated by independent molecular methods such as qRT-PCR, and the chlorophyll estimates are based on spectrophotometry of crude extracts and should be confirmed chromatographically. Protein-level correlates of the annotation patterns remain unconfirmed, particularly for *La. punctata*, where extensive post-transcriptional regulation has been reported in related *Lemna* species [15]; matched proteomics and metabolomics would help validate functional relevance. Although our study provides only baseline transcriptomic resources and species-level physiological comparisons, these may help generate hypotheses for future work. For example, the stable fresh weight of *L. minor* across media, the relatively high protein concentration of *La. punctata* and *L. aequinoctialis* on basal MS, and the high fresh weight of *L. gibba* and *L. aequinoctialis* on basal MS suggest candidate traits for evaluation in longer-term growth trials. Any application in feed or food production [17], phytoremediation [19], or biorefinery would require experimental validation under application-relevant conditions, including multiple accessions, dry-weight-based composition, longer cultivation periods, and scale-up studies. As additional Lemnaceae genomes and phylogenomic resources become available [12], it will also be possible to test whether the expression differences reported here reflect phylogeny, ecology, or lineage-specific gene family changes.

## 4. Materials and Methods

### 4.1. Plant Material and Growth Conditions

Five duckweed species, *L. minor* (RDSC 7295), *L. japonica* (RDSC 5697), *L. gibba* (RDSC 8124), *La. punctata* (RDSC 5635), *and L. aequinoctialis* (RDSC 8224), were procured from Rutgers Duckweed Stock Cooperative (RDSC, Rutgers, New Brunswick, NJ, USA) and maintained axenically in half-strength MS medium (PhytoTech Labs, Lenexa, KS, USA) under a 16 h light/8 h dark photoperiod; 25 ± 1 °C; light intensity 80 μmol m^−2^ s^−1^ before experiments. For RNA-seq, each species was grown on basal MS medium (MS basal salts with 10 g L^−1^ sucrose) under the conditions above for 7 days, with three independent biological replicates (separate culture vessels) per species (15 samples in total). Whole fronds were harvested at 6 h after the onset of the light period, blotted dry, flash-frozen in liquid nitrogen, and stored at −80 °C. For physiological assays, healthy fronds from 7-day-old stock cultures were transferred 3 mL of each of four media in 12 wells plate at an initial inoculum of five plants of three fronds each per well: Basal MS, Hoagland salt solution (Hoagland’s No. 2 basal salt mixture; Caisson Labs, Smithfield, UT, USA), Gamborg B5 medium (B5 basal salts with vitamins; PhytoTech Labs, Lenexa, KS, USA), and nitrogen-free MS (MS basal salts without nitrogen, MS–N; bioWorld, plantMedia, Dublin, OH, USA). The nominal composition of each medium is given in Table 1. Plants were grown for 7 days under the conditions above. Three independent cultures (biological replicates) were established per species × medium combination.

### 4.2. RNA Extraction and Transcriptome Sequencing

Total RNA was extracted from approximately 100 mg fresh weight of frond tissue using the GeneJET Plant RNA Purification Kit (Thermo Scientific, Waltham, MA, USA) according to the manufacturer’s instructions. RNA quality and integrity were assessed using an Agilent Bioanalyzer 2100 (Agilent Technologies, Santa Clara, CA, USA); samples with RNA Integrity Number (RIN) ≥ 8.0 were used for library preparation. Paired-end RNA-seq libraries (PE150) were prepared using the DNBSEQ Eukaryotic Strand-specific mRNA library and sequenced on the DNBSEQ platform at INNOMICS Inc. (Sunnyvale, CA, USA). The libraries were prepared as poly(A)+ mRNA enrichment using oligo(dT) beads, RNA fragmentation, random-primer reverse transcription and second-strand cDNA synthesis with dUTP in place of dTTP, followed by end repair, adenylation, adapter ligation, PCR amplification, circularization and DNA nanoball preparation. The reads were preprocessed with SOAPnuke v2.2.2 and supplied as Phred + 33 FASTQ files [48].

### 4.3. De Novo Transcriptome Assembly and Deduplication

The filtered paired-end reads were further processed with fastp v1.1.0 [49] using its default trimming and filtering settings, and quality-control summaries from all fastp reports were aggregated with MultiQC v1.33 [50]. For each sample, Table S11 reports read counts (distinguishing individual reads from read pairs), read and base retention, the percentage of Q30 bases, and mapping statistics. De novo transcriptomes were assembled separately for each species with Trinity v2.15.2 [51], combining the paired reads of its three biological replicates. In silico read normalization used a k-mer length of 25, a maximum coverage of 200, a minimum coverage of 1, and a maximum coefficient of variation of 10,000, with paired reads normalized together. The minimum contig length was 200 bp, the executable default, as confirmed in all run logs. Deduplication was evaluated as a separate assembly quality comparison. Redundant transcripts were clustered with CD-HIT-EST v4.8.1 [52,53] at 99% local sequence identity and at least 90% alignment coverage of the shorter sequence, using a word length of 10 and the accurate clustering mode (complete command in Table S12). Completeness of the assemblies before and after deduplication was assessed with BUSCO v6.0.0 [27,28] against the Viridiplantae (odb12; 822 genes), Embryophyta (odb12; 2026 genes), and Liliopsida (odb12; 2821 genes) datasets. Protein prediction, orthogroup inference, and expression quantification used the original, non-deduplicated Trinity assemblies.

### 4.4. Orthogroup Inference, Expression Quantification, PCA, and Correlation Analyses

Proteins were predicted from each non-deduplicated Trinity assembly with TransDecoder v5.5.0 [54] (LongOrfs followed by Predict), and the longest predicted protein of each Trinity gene was retained as its representative. Orthogroups were inferred with OrthoFinder v2.5.5 [24] from the representative proteomes. The OrthoFinder run comprised the five study species, and OG membership for the five study species was taken from this run. Of the 18,484 OGs in the expression matrix, 18,390 contained at least one representative protein from the five study species. Because the proteomes were derived from transcriptome assemblies, the presence of an OG in a species denotes detection in that species’ assembled transcriptome, not genomic presence or absence.

Transcript abundance was quantified with Salmon v1.10.3 [55] against species-specific dense indices built from the non-deduplicated Trinity assemblies (k-mer length 31; no decoy sequences), using automatic library-type detection and mapping validation. Quantification used the provider-filtered paired reads directly rather than the fastp output. Sequence- and GC-bias correction were disabled, and no bootstrap replicates were drawn. All 15 samples were classified as library type IU. Mapping rates, calculated as mapped read pairs divided by processed read pairs, are reported in Table S11, and index and quantification commands and metadata are documented in Table S12. Protein identifiers were mapped to their source transcripts by removing the terminal protein suffix, and the expression of each OG in each sample was calculated as the sum of the TPM values of the representative transcripts assigned to it. OG expression values therefore describe representative-transcript abundance rather than the sum of all transcript isoforms.

For correlation analyses, OGs with TPM ≥ 1 in at least 3 of the 15 samples were retained (15,151 of 18,390 OGs). Pearson correlation coefficients between samples were calculated across these OGs after log_2_(TPM + 1) transformation. Species-level correlations used the same OG set, with TPM averaged across the three biological replicates of each species before log_2_(mean TPM + 1) transformation. No distance transformation was applied. Only unique off-diagonal correlations were summarized, giving 15 within-species sample pairs, 90 between-species sample pairs, and 10 species-mean pairs; medians and first and third quartiles were calculated separately for each set using linearly interpolated sample quantiles.

The difference between within-species and between-species sample correlations was tested with the statistic T = median(rwithin) − median(rbetween). Species labels were permuted among the 15 samples 10,000 times while preserving five groups of three samples (NumPy PCG64 generator; seed 20260927). The correlations themselves were held fixed, and the within- and between-species pair sets were redefined in each permutation. The one-sided Monte Carlo *p* value was calculated as (b + 1)/(10,000 + 1), where b is the number of permuted statistics at least as large as the observed statistic [56]; the smallest attainable value is therefore approximately 1.0 × 10^−4^. Permuting sample labels avoids treating the 105 correlated sample pairs as independent observations; the test assumes exchangeability of sample labels under the null hypothesis.

PCA was performed on log_2_(TPM + 1) values of the 18,484-OG matrix. The 5000 OGs with the highest variance across the 15 samples were centered and scaled to unit population standard deviation (scikit-learn StandardScaler) [57] before PCA, and all 15 samples were plotted. This PCA input set is distinct from the filtered OG set used for the correlation analyses. For the expression heatmap (Figure 2C), the 100 OGs with the highest variance across the five log_2_-transformed species-mean TPM profiles were selected from the correlation OG set and standardized within each OG across species using the sample standard deviation; rows were ordered by hierarchical clustering (Euclidean distance, average linkage), and the species column order was fixed.

### 4.5. Species Signature Scores and Balanced Internal Validation

The signature analysis assessed whether a contrast-based marker-selection and scoring procedure retained its species pattern in withheld samples, conditional on the existing expression matrix. Marker selection and evaluation were separated to avoid assessing a contrast in the samples used to select its markers [58,59]. We enumerated all 35 = 243 splits that contain one held-out sample from each species. Each split comprised 10 training samples (two per species) and five held-out samples, so that each species contributed equally to training and score calibration, and each original sample appeared in 81 test sets. The overlapping splits were not treated as independent experimental replicates.

Three OG universes were defined from the existing OG assignments: all 18,390 OGs represented in at least one study species (“all retained”); the 8040 OGs represented in all five species (“shared”); and the 2941 OGs with exactly one retained representative protein in each of the five species (“single representative”). The shared set was used for the primary analysis because it restricts the comparison to OGs represented in every species. Within each training set and universe, the primary expression filter required TPM ≥ 1 in at least two training samples, which corresponds to the two training replicates per species and allows OGs detected in both training samples of a single species to remain eligible. A threshold of at least three training samples was analyzed as a sensitivity check.

For each eligible OG, TPM was averaged within each training species and transformed as log_2_(mean TPM + 1). The focal-species contrast was the transformed focal-species mean minus the equally weighted mean of the other four species. OGs were ranked by decreasing contrast, with the OG identifier resolving ties, and the 10, 30, 50, 100, and 200 highest-ranked OGs were selected separately for each species. All combinations of three universes, two expression thresholds, and five signature sizes were analyzed as 30 sensitivity configurations. For each signature, the raw score of a sample was the mean log_2_(TPM + 1) across the selected OGs. Each score was centered on the mean of the 10 training-sample scores for that signature and divided by their sample standard deviation; the calibrated score expresses a sample’s position relative to the training distribution and is neither a probability nor a biological effect size. For each held-out sample, the margin was calculated as the cognate score minus the largest of the four non-cognate scores, so that a positive margin indicates that the cognate signature scored highest under that split-specific calibration. No training-score standard deviation fell below the pre-specified stopping threshold of 10–12, and no exact ties for the highest score occurred.

Scores and margins were summarized for each of the 15 samples over its 81 held-out appearances. Marker-selection frequency was the fraction of the 243 training splits in which an OG was selected. Marker stability was described by the Jaccard index (size of the intersection divided by size of the union) for all 29,403 unordered pairs of selected-marker sets per species and configuration, summarized as medians and first and third quartiles of these dependent values. Overlap with each original all-sample top-30 list was reported separately as a descriptive comparison. The 1215 held-out sample appearances per configuration were not regarded as independent observations, and no binomial confidence intervals or signature-specific permutation tests were calculated. This analysis evaluates the selection-and-scoring procedure; it is not an independent evaluation of the original fixed marker lists, and selection frequencies do not validate any post hoc consensus list. All validation steps used the precomputed expression matrix: the species-specific references had been assembled from all three samples of each species, reads had been quantified against the reference of their known species, and references, OG assignments, and abundance estimates were not rebuilt within splits. Signature scores, margins, marker lists, selection frequencies, and Jaccard summaries are provided in Table S3. Analyses used Python 3.12.2, NumPy 1.26.4 [60], pandas 2.2.2, SciPy 1.13.1 [61], and scikit-learn 1.4.2 [57].

### 4.6. Functional Annotation and Annotation Composition

Representative proteins used for OG inference were annotated with eggNOG-mapper v2.1.13 [62,63] in DIAMOND mode, against the eggNOG 5.0.2 annotation database [32]. Searches used DIAMOND v2.0.15 in sensitive mode with iterative searching and an E-value threshold of 0.001; alignments within 3% of the best score for each query were reported, and no additional sequence-identity or alignment-coverage cutoff was applied. The Liliopsida taxonomic scope controlled annotation transfer within eggNOG-mapper; it did not restrict the DIAMOND database search to plant sequences. Complete commands, output fields, and version records are provided in Table S12. GO terms, KO identifiers, KEGG pathways, and KEGG modules were analyzed from the eggNOG-mapper output [64,65]. Annotations were aggregated as the union of member-protein annotations within each species and OG, and each OG was counted once per term. Alternate GO identifiers and unambiguous replaced_by chains were resolved; obsolete GO terms without an unambiguous replacement, unknown terms, and KEGG identifiers absent from the retrieved lists were excluded, with their original records retained in the analysis files. Composition was calculated as the fraction of annotation-eligible OGs carrying each term, with the denominator defined separately for each species and annotation class (and, for GO, for each namespace). These are binary annotation counts, not expression measurements or expression-weighted counts, and the fractions do not sum to one because an OG can carry several annotations. Terms were ranked by the sample standard deviation of their fractions across the five species (term identifier breaking ties). Selected fractions were centered and divided by their sample standard deviation across species (row z-scores), rows were ordered by average-linkage hierarchical clustering with Euclidean distance and optimal leaf ordering, and species columns were kept in a fixed order; the color scale (−2 to 2) contains every displayed value. For the GO display, candidate terms required a shortest is_a depth of at least 4, at least five distinct OGs across species, and a maximum namespace-specific fraction of 0.20, so that broad and root terms were removed; candidates were then considered in order of decreasing variability, a term was skipped if its OG membership had a Jaccard similarity > 0.85 with an already selected term, and the first 24 terms were displayed. The KEGG pathway display omitted global and overview maps, human-disease and drug-development maps, and maps describing animal organismal or animal/yeast reference contexts other than designated plant maps; the 20 most variable eligible pathways and 18 most variable current modules were displayed. Composition summarizes one assembled annotation repertoire per species, so no replicate-level error bars or hypothesis tests are implied. Annotation rates per species are reported in Table S11.

### 4.7. Species-Specific Overrepresentation Analysis

For each species, the foreground comprised OGs detected only in the focal species among the five study species (species-restricted OGs), intersected with the annotation-specific background. The background comprised all OGs present in that species with at least one valid annotation in the tested class. GO biological process, molecular function, and cellular component, and KEGG KO, pathway, and module annotations were analyzed as separate classes. One-sided hypergeometric tests [34] evaluated every term with 5–500 background OGs, including terms with no foreground hits; terms outside this size range were untested rather than non-significant. *p* values were adjusted with the Benjamini–Hochberg procedure within each species × annotation-class family [66]. Fold enrichment was calculated as (k/*n*)/(K/N), where k is the number of foreground OGs annotated with the term, *n* the foreground size, K the number of background OGs annotated with the term, and N the background size; a foreground of size zero received *p* = 1 and an undefined fold enrichment. FDR ≤ 0.05 and FDR ≤ 0.10 were pre-specified as the primary and exploratory significance thresholds, respectively.

For the exploratory display in Figure 3B, up to three tested GO terms with k > 0 were selected per species, ordered by increasing FDR, increasing unadjusted *p* value, decreasing fold enrichment, and increasing GO identifier. For Figure 4C,D, up to two tested terms with k > 0 were selected per species separately for KO and pathway analyses using the same ordering, and composition-display exclusions were not applied. No FDR cutoff was applied to these display selections, and the selection rules did not alter the tested terms, *p* values, or correction families. Point color represents −log10(FDR), and point area is proportional to max(fold enrichment, 1). All displayed terms have FDR > 0.10. Complete results, including k, *n*, K, N, *p*, FDR, and fold enrichment for all tested terms, are provided in Table S13.

### 4.8. Physiological Assays

Each assay was performed on three biological replicates per species × medium combination, and each biological replicate was measured in three technical replicates (separate aliquots of the same extract or assay wells), which were averaged to give one value per biological replicate. For protein, peroxidase, and chlorophyll, values measured in the assay aliquot were scaled to the total volume of the tissue lysate or extract (1 mL) and divided by the fresh weight of the extracted tissue, so that all traits are expressed per mg FW on the same basis. Fresh weight was determined after harvesting all fronds of each culture, blotting them dry on filter paper, and weighing them immediately on an analytical microbalance.

Protein concentration was determined with the Bradford assay [67]. Fresh tissue (20 mg) was homogenized in 1 mL of 1X phosphate-buffered saline and centrifuged at 13,000× *g* for 10 min at 4 °C; 50 μL of supernatant was mixed with 50 μL of Bradford reagent, and absorbance was read at 595 nm after 30 min. Protein was quantified against a bovine serum albumin standard curve (0–100 μg mL^−1^), multiplied by the lysate volume, and expressed as μg protein mg^−1^ FW.

Glucose concentration was determined by the glucose oxidase–peroxidase/o-dianisidine method [68,69]. Fresh tissue (20 mg) was homogenized in 1 mL of ice-cold 50 mM potassium phosphate buffer (pH 7.0) and centrifuged at 13,000× *g* for 10 min at 4 °C. An aliquot of the supernatant (20 μL) was mixed with 180 μL of reagent containing glucose oxidase (100 U mL^−1^), horseradish peroxidase (10 U mL^−1^), and o-dianisidine (1 mg mL^−1^) in 50 mM potassium phosphate buffer (pH 7.0) and incubated at 37 °C for 30 min. In this reaction, glucose oxidase converts glucose to gluconic acid and H_2_O_2_, and peroxidase uses the H_2_O_2_ to oxidize o-dianisidine to a colored product. Absorbance was read at 540 nm. Glucose was quantified against a D-glucose standard curve (0–1000 μg mL^−1^) prepared in the same buffer and processed in parallel with the samples, multiplied by the lysate volume, and expressed as μg glucose mg^−1^ FW.

Guaiacol peroxidase activity was assayed spectrophotometrically [67]. Tissue (20 mg) was homogenized in 1 mL of ice-cold 50 mM potassium phosphate buffer (pH 7.0) and centrifuged at 13,000× *g* for 10 min at 4 °C. The reaction mixture contained 50 mM potassium phosphate buffer (pH 7.0), 15 mM guaiacol, 5 mM H_2_O_2_, and 20 μL of crude enzyme extract; the reaction was started by adding H_2_O_2_, and the increase in absorbance at 470 nm was recorded kinetically for 10 min at 1 min intervals at 25 °C. One unit (U) of peroxidase activity was defined as the amount of enzyme causing an increase in A470 of 0.01 per min, and activity in the assayed aliquot was scaled to the total lysate volume and expressed as U mg^−1^ FW.

Chlorophylls were extracted from 20 mg of fresh tissue in 1 mL of 80% (*v*/*v*) acetone at 4 °C in darkness for 16 h, and extracts were clarified by centrifugation at 13,000× *g* for 10 min. Absorbance was measured at 663 and 646 nm, and concentrations in the extract (μg mL^−1^) were calculated with the equations of Lichtenthaler and Wellburn for 80% acetone [46]: chlorophyll a = 12.21 A_663_ − 2.81 A_646_; chlorophyll b = 20.13 A_646_ − 5.03 A_663_. Concentrations were converted to μg mg^−1^ FW by multiplying by the extract volume and dividing by the tissue fresh weight.

### 4.9. Statistical Analysis

For each trait, biological-replicate values (*n* = 3 per group; technical replicates averaged) were analyzed by two-way ANOVA with species, medium, and their interaction as fixed effects in GraphPad Prism v10.0 (GraphPad Software, Boston, MA, USA). Cell means were then compared with Tukey’s multiple comparisons test within each species (six comparisons per family) and within each medium (ten comparisons per family), with the correction applied separately within each of the nine families. Residual normality and homogeneity of variance were assessed by Q–Q plots of residuals and the Brown–Forsythe test. F statistics, degrees of freedom, exact *p* values, and partial eta-squared (η^2^*_p_*) effect sizes are reported in Table 2. Figure 5 shows the comparisons of each medium with basal MS within each species. Significance thresholds were ns (*p* ≥ 0.05), * (*p* < 0.05), ** (*p* < 0.01), *** (*p* < 0.001), and **** (*p* < 0.0001).

## 5. Conclusions

This study provides a comparative description of baseline transcriptomes and nutrient-medium physiological responses in five duckweed species, *L. minor*, *La. punctata*, *L. japonica*, *L. gibba*, and *L. aequinoctialis*. Under a common growth condition, the five species showed distinct and reproducible orthogroup expression profiles, and species signatures assigned held-out replicates correctly across all balanced validation splits. GO and KEGG annotation composition differed among species, but species-specific enrichment tests were not statistically significant, so the annotation patterns are presented as hypotheses for targeted validation rather than as evidence of species-specific pathway activity. In a separate experiment, species differed in their physiological responses to four nutrient media; nitrogen-free MS lowered protein and chlorophyll a and increased glucose and chlorophyll b in all species, but the media differed in several components, and we did not obtain transcriptomic and physiological data under matched conditions. These datasets provide a resource for comparative Lemnaceae biology and may help prioritize species and traits for future experiments, including matched transcriptomic–physiological studies, multiple accessions, longer cultivation periods, and validation under application-relevant conditions.

## Figures and Tables

**Table 1:** Nominal macronutrient composition of the four media used for physiological assays ^a^.

Component (mM Unless Stated)	Basal MS	Hoagland ^b^	Gamborg’s B5	MS–N ^c^
Total N	60.0	15.0	26.8	0
NO_3_^−^/NH_4_^+^	39.4/20.6	14.0/1.0	24.7/2.0	0/0
K^+^	20.0	6.0	24.7	1.25 ^c^
Phosphate	1.25	1.0	1.1	1.25
Ca^2+^	3.0	4.0	1.0	3.0
Mg^2+^	1.5	2.0	1.0	1.5
Iron source (Fe, mM)	FeSO_4_–Na_2_EDTA (0.10)	Fe-tartrate	FeSO_4_–Na_2_EDTA (0.10)	FeSO_4_–Na_2_EDTA (0.10)
Vitamins	yes	no	yes	no
Sucrose (g L^−1^)	10	10	10	10
Supplier (cat. no.)	PhytoTech (M519)	Caisson (HOP01)	PhytoTech (G398)	Plantmedia (30630200)

^a^ Nominal concentrations calculated from the standard published formulations of each basal salt mixture; micronutrient compositions also differ among media and are listed in the manufacturers’ formulation sheets. ^b^ Values for Hoagland’s No. 2 basal salt formulation. ^c^ MS–N omits NH_4_NO_3_ and KNO_3_ and is without vitamins; no compensating potassium salt is included, so K^+^ is supplied only by KH_2_PO_4_.

**Table 2:** Two-way ANOVA of physiological traits (species × medium; *n* = 3 biological replicates per group; residual df = 40). Values are from GraphPad Prism.

Trait	Species F_4,40_	*p*	η^2^*_p_*	Medium F_3,40_	*p*	η^2^*_p_*	S × M F_12,40_	*p*	η^2^*_p_*
Fresh weight	1038	<0.0001	0.990	247.3	<0.0001	0.949	42.07	<0.0001	0.927
Protein	1.481	0.2260	0.129	136.1	<0.0001	0.911	5.599	<0.0001	0.627
Glucose	104.2	<0.0001	0.912	90.32	<0.0001	0.871	5.667	<0.0001	0.630
Peroxidase	1429	<0.0001	0.993	631.5	<0.0001	0.979	112.8	<0.0001	0.971
Chlorophyll a	79.81	<0.0001	0.889	162.2	<0.0001	0.924	5.953	<0.0001	0.641
Chlorophyll b	115.1	<0.0001	0.920	192.7	<0.0001	0.935	16.51	<0.0001	0.832

F, F statistic with numerator and denominator degrees of freedom; *p*, *p* value; η^2^*_p_*, partial eta-squared; S × M, species × medium interaction.

## Data Availability

The data presented in this study (GSE349341) are available in the NCBI Gene Expression Omnibus (GEO) database.

## Supplementary Materials

The following supporting information can be downloaded at: https://www.mdpi.com/article/10.3390/ijms27219681/s1.
